# Contemporary Burden and Correlates of Symptomatic Paroxysmal Supraventricular Tachycardia

**DOI:** 10.1161/JAHA.118.008759

**Published:** 2018-07-07

**Authors:** Alan S. Go, Mark A. Hlatky, Taylor I. Liu, Dongjie Fan, Elisha A. Garcia, Sue Hee Sung, Matthew D. Solomon

**Affiliations:** ^1^ Division of Research Kaiser Permanente Northern California Oakland CA; ^2^ Departments of Epidemiology, Biostatistics and Medicine University of California, San Francisco CA; ^3^ Department of Health Research and Policy Stanford University Stanford CA; ^4^ Department of Medicine (Cardiovascular Medicine) Stanford University Stanford CA; ^5^ Department of Cardiac Electrophysiology Kaiser Permanente Santa Clara Medical Center Santa Clara CA; ^6^ Department of Cardiology Kaiser Permanente Oakland Medical Center Oakland CA

**Keywords:** elderly, epidemiology, incidence, sex differences, supraventricular tachycardia, Arrhythmias, Epidemiology, Risk Factors

## Abstract

**Background:**

Contemporary data about symptomatic paroxysmal supraventricular tachycardia (PSVT) epidemiology are limited. We characterized prevalence and correlates of symptomatic PSVT within a large healthcare delivery system and estimated national PSVT burden.

**Methods and Results:**

We identified adults with an encounter for potential PSVT between 2010 and 2015 in Kaiser Permanente Northern California, excluding those with prior known atrial fibrillation or atrial flutter. We adjudicated medical records, ECGs, and other monitoring data to estimate positive predictive values for targeted *International Classification of Diseases (ICD), 9th and 10th Revisions* codes in inpatient, emergency department, and outpatient settings. Combinations of diagnosis codes and settings were used to calculate PSVT prevalence, and PSVT correlates were identified using multivariable regression. We estimated national rates by applying prevalence estimates in Kaiser Permanente to 2010 US Census data. The highest positive predictive values included codes for “PSVT” in the emergency department (82%), “unspecified cardiac dysrhythmia” in the emergency department (27%), “anomalous atrioventricular excitation” as a primary inpatient diagnosis (33%), and “unspecified paroxysmal tachycardia” as a primary inpatient diagnosis (23%). Prevalence of symptomatic PSVT was 140 per 100 000 (95% confidence interval, 100–179) and was higher for individuals who were older, women, white or black, or who had valvular heart disease, heart failure, diabetes mellitus, lung disease, or prior bleeding. We estimate the national prevalence of symptomatic PSVT to be 168 per 100 000 (95% confidence interval, 120–215).

**Conclusions:**

Selected diagnostic codes in inpatient and emergency department settings may be useful to identify symptomatic PSVT episodes. We project that at least 0.168% of US adults experience symptomatic PSVT, and certain characteristics can identify people at higher risk.

**Clinical Perspective**

**What Is New?**

- This study provides contemporary, community‐based information on the accuracy of diagnostic codes to identify episodes of symptomatic paroxysmal supraventricular tachycardia and on the national burden of symptomatic paroxysmal supraventricular tachycardia.

**What Are the Clinical Implications?**

- Several patient subgroups (older age, female, black race, Hispanic ethnicity, valvular heart disease, heart failure, diabetes mellitus, lung disease, bleeding history) experience a higher burden of symptomatic episodes of paroxysmal supraventricular tachycardia.

## Introduction

Paroxysmal supraventricular tachycardia (PSVT) other than atrial fibrillation or atrial flutter includes a heterogeneous set of conditions involving abnormal automaticity (eg, focal ectopic atrial tachycardia, multifocal atrial tachycardia, and junctional tachycardia) and abnormal reentry (eg, atrioventricular nodal reentrant tachycardia and atrioventricular reentrant tachycardia with accessory pathways). PSVTs are managed using a variety of approaches depending on the underlying mechanism and the clinical stability and frequency of episodes.^1^ PSVTs can have a negative effect on patient quality of life by causing symptoms during episodes (eg, palpitations, chest pain, dizziness, syncope, and shortness of breath), by imposing restrictions on patient activities, and by leading to medication‐related side effects.

Despite symptomatic PSVTs being considered the most frequent indication for catheter ablation procedures, reliable, contemporary data on the population burden of PVSTs are quite limited. The most frequently quoted estimate of PSVT prevalence in the United States is based on a study from the Marshfield Clinic, in central Wisconsin, using data from January 1979 through June 1993.^2^ After standardizing to the age and sex distribution in the US adult population, the investigators projected ≈570 000 prevalent cases and ≈89 000 incident cases per year of PSVT in the United States.^2^ This study was limited by its relatively small sample size, inclusion of a selected midwestern community that is not representative of the broad racial/ethnic diversity in the United States, and data that no longer reflect contemporary practice. During the past 25 years, there have been increases in heart failure, hypertension, diabetes mellitus, and obesity, all of which may have affected the prevalence of PSVT. In addition, this study relied on *International Classification of Diseases* (*ICD*) diagnostic codes found in electronic health records and billing claims—data that are readily available from health systems—but also showed low accuracy for the evaluated codes in validation studies. In contrast, population estimates based on highly selected patients referred for catheter ablation or other electrophysiology procedures benefit from more accurate patient identification but are likely biased by omitting the large majority of medically managed PSVT patients.

Patient factors associated with the development of symptomatic PSVT are also poorly understood. Data from Marshfield Clinic suggested that female sex, older age, and the presence of clinical cardiovascular disease were each associated with higher risk of presenting with PSVT.^2^ In addition, it is possible that atrioventricular nodal reentrant tachycardia and atrioventricular reentrant tachycardia may be more common in younger individuals.^2^ The relative frequency of PSVT mediated by an accessory pathway may also be lower with increasing age.^2^

To address the limitations of existing data on contemporary PSVT, we examined its epidemiology and risk factors among a large, community‐based population with broad demographic diversity and used these data to estimate the national burden of symptomatic PSVT.

## Methods

The data, analytic methods, and study materials will not be made available to other researchers for purposes of reproducing the results or replicating the procedure.

### Source Population

The source population for the PREEMPT (Population‐Based Risk and Epidemiology of Paroxysmal Supraventricular Tachycardias) study included members of Kaiser Permanente Northern California (KPNC), an integrated healthcare delivery system that provides comprehensive medical care to >4.2 million people. The KPNC membership is highly representative of the local and statewide population with respect to age, sex, race/ethnicity, and socioeconomic status.^3^, ^4^, ^5^

The study was approved by the KPNC institutional review board, and a waiver of informed consent was obtained given the nature of the study.

### Identification of PSVT Episodes

We searched electronic health records from January 1, 2010, and December 31, 2015, for diagnostic codes suggestive of symptomatic PSVT encounters in the inpatient, emergency department and outpatient clinic settings. We identified encounters with *ICD‐9* codes 426.7 (anomalous atrioventricular excitation), 427.0 (PSVT), 427.2 (paroxysmal tachycardia, unspecified), 427.61 (supraventricular premature beats), 427.89 (other specified cardiac dysrhythmias), 427.9 (cardiac dysrhythmia, unspecified), 785.0 (tachycardia, unspecified), and 785.1 (palpitations).^2^ After October 1, 2015, when KPNC adopted *ICD‐10* codes, we examined encounters for I45.6 (preexcitation syndrome), I47.1 (supraventricular tachycardia), I47.9 (paroxysmal tachycardia, unspecified), I49.1 (atrial premature depolarization), I49.8 (other specified cardiac arrhythmias), I49.9 (cardiac arrhythmia, unspecified), R00.0 (tachycardia, unspecified), and R00.2 (palpitations).

### Validation of PSVT Diagnostic Codes

We assessed the validity of diagnostic codes for potential PSVT episodes by reviewing a stratified random sample of up to 150 electronic medical records for each diagnostic code; these were further stratified by healthcare setting (inpatient, emergency department, and outpatient).

Physician adjudicators reviewed relevant medical records, including 12‐lead ECG, reports from long‐term rhythm monitoring recorders (eg, 24‐hour Holter, event loop recorder, patch‐based electrocardiographic monitoring solutions), electrophysiology procedure reports, and physician progress notes. A symptomatic PSVT episode required evidence of >30‐second duration during the specific clinical encounter documented in any setting and/or captured with ECG monitoring or electrophysiology procedure; if available, the specific type of PSVT was documented using standardized criteria. A documented prior history of PSVT was not considered a valid outcome.

### Patient Characteristics

We collected information on patient age, sex and self‐reported race/ethnicity from health plan databases. The presence of chronic kidney disease was based on an estimated glomerular filtration rate <60 mL/min per 1.73 m^2^ using the Chronic Kidney Disease Epidemiology Collaboration (CKD‐EPI) estimating equation.^6^ Diabetes mellitus status was determined based on *ICD* diagnostic codes, laboratory results, or receipt of diabetes mellitus–specific medications within a regional diabetes mellitus registry.^7^ Hypertension was determined based on >2 outpatient diagnoses or ≥1 diagnosis and receipt of antihypertensive therapy.^8^ Coronary heart disease was defined as a history of hospitalization for acute myocardial infarction or the receipt of coronary artery bypass surgery or percutaneous coronary intervention.^8^, ^9^ Heart failure was defined as hospitalization with a primary discharge diagnosis of heart failure or ≥3 outpatient encounters coded for heart failure using relevant *ICD* codes, with at least 1 visit being to a cardiologist.^10^

### Statistical Analysis

All analyses were performed using SAS, version 9.3 (SAS Institute). In our validation study, we calculated the positive predictive value (PPV) and associated 95% confidence interval (CI) for each diagnostic code and clinical setting combination and reported results if they met the following criteria: total number of reviewed encounters >10 for the diagnostic code/setting combination and having a PPV >15% for the diagnostic code/setting combination. Using our final PSVT episode identification algorithm, we next calculated the period prevalence of symptomatic PSVT episodes (per 100 000) with associated CIs among adults aged ≥18 years in the KPNC population. To calculate incidence of symptomatic PSVT episodes, we repeated analyses after excluding any people in the source population who met diagnostic criteria for PSVT up to 4 years before study entry. For both prevalence and incidence during the study period, we conducted subgroup analyses by age, sex, race/ethnicity, and the presence or absence of chronic kidney disease, diabetes mellitus, hypertension, coronary heart disease, and heart failure.

Finally, to estimate the national burden of PSVT episodes, we calculated age‐ and sex‐adjusted prevalence of symptomatic PSVT episodes in the United States by applying the KPNC age‐ and sex‐stratified prevalence estimates to US Census data from 2010.

## Results

### Accuracy of Diagnostic Codes for PSVT in Different Care Settings

We adjudicated a sample of 1238 potential symptomatic PSVT episodes across inpatient, emergency department, and outpatient visit settings that met our eligibility criteria but subsequently excluded 418 encounters with combinations of code and care settings that could not provide reliable estimates. Among the remaining 820 encounters, 223 (27%; 95% CI, 24%–29%) were confirmed to be episodes of symptomatic PSVT, with wide variation in the PPV across different combinations of codes and settings (Table 1). The highest PPVs were for the *ICD‐9/10* codes for “PSVT” recorded in the inpatient or emergency department setting, followed by “anomalous atrioventricular excitation” in the inpatient or emergency department setting and “unspecified cardiac dysrhythmia” in the emergency department setting (Table 1).

**Table 1:** PPV of Diagnostic Codes for Symptomatic PSVT Episodes (2010–2015)

*ICD* Code	Description	Setting	Charts Reviewed	Confirmed PSVT Episode	PPV, %	95 (CI)
426.7	Anomalous atrioventricular excitation	Inpatient primary	12	4	33.3	9.9–65.1
		Inpatient secondary	2	1	50.0	1.3–98.7
		Emergency	55	18	32.7	20.7–46.7
		Outpatient	40	4	10.0	2.8–23.7
I45.6	Preexcitation syndrome	Inpatient primary	0	N/A	N/A	N/A
		Inpatient secondary	0	N/A	N/A	N/A
		Emergency	4	0	0.0	0–60.2
		Outpatient	16	1	6.3	0.2–30.2
427.0	PSVT	Inpatient primary	36	22	61.1	43.5–76.9
		Inpatient secondary	36	12	33.3	18.6–51.0
		Emergency	37	27	73.0	55.9–86.2
		Outpatient	36	4	11.1	3.1–26.1
I47.1	Supraventricular tachycardia	Inpatient primary	20	9	45.0	23.1–68.5
		Inpatient secondary	3	1	33.3	0.8–90.6
		Emergency	89	73	82.0	72.5–89.4
		Outpatient	39	9	23.1	11.1–39.3
427.2	Paroxysmal tachycardia, unspecified	Inpatient primary	21	3	14.3	3.0–36.3
		Inpatient secondary	11	1	9.1	0.2–41.3
		Emergency	65	15	23.1	13.5–33.3
		Outpatient	44	0	0.0	0–97.5
I47.9	Paroxysmal tachycardia, unspecified	Inpatient primary	1	0	0.0	0–97.5
		Inpatient secondary	0	N/A	N/A	N/A
		Emergency	1	0	0.0	0–97.5
		Outpatient	16	1	6.3	0.2–30.2
427.61	Supraventricular premature beats	Inpatient primary	3	1	33.3	0.8–90.6
		Inpatient secondary	7	0	0.0	0.0–41.0
		Emergency	25	1	4.0	0.1–20.4
		Outpatient	18	0	0.0	0.0–18.5
I49.1	Atrial premature depolarization	Inpatient primary	0	N/A	N/A	N/A
		Inpatient secondary	0	N/A	N/A	N/A
		Emergency	3	0	0.0	0.0–70.8
		Outpatient	37	2	5.4	0.7–18.2
427.89	Other specified cardiac dysrhythmias	Inpatient primary	10	0	0.0	0.0–30.8
		Inpatient secondary	17	0	0.0	0.0–19.5
		Emergency	38	5	13.2	4.4–28.1
		Outpatient	20	0	0.0	0.0–16.8
I49.8	Other specified cardiac arrhythmias	Inpatient primary	0	N/A	N/A	N/A
		Inpatient secondary	0	N/A	N/A	N/A
		Emergency	0	N/A	N/A	N/A
		Outpatient	11	0	0.0	0–28.5
427.9	Cardiac dysrhythmia, unspecified	Inpatient primary	50	4	8.0	2.3–19.6
		Inpatient secondary	9	0	0.0	0.0–33.6
		Emergency	37	10	27.0	13.8–44.1
		Outpatient	19	1	5.3	0.1–26.0
I49.9	Cardiac arrhythmia, unspecified	Inpatient primary	6	1	16.7	0.4–64.1
		Inpatient secondary	1	0	0.0	0–97.5
		Emergency	3	1	33.3	0.8–90.6
		Outpatient	34	2	5.9	0.7–19.7
785.0	Tachycardia, unspecified	Inpatient primary	37	0	0.0	0.0–9.5
		Inpatient secondary	19	0	0.0	0.0–9.5
		Emergency	16	1	6.3	0.2–30.2
		Outpatient	22	0	0.0	0.0–15.4
R00.0	Tachycardia, unspecified	Inpatient primary	18	0	0.0	0–18.5
		Inpatient secondary	6	1	16.7	0.4–64.1
		Emergency	16	2	12.5	1.6–38.3
		Outpatient	17	0	0.0	0.0–19.5
785.1	Palpitations	Inpatient primary	37	0	0.0	0.0–10.6
		Inpatient secondary	15	2	13.3	1.7–40.5
		Emergency	22	0	0.0	0.0–15.4
		Outpatient	39	1	2.6	0.1–13.2
R00.2	Palpitations	Inpatient primary	3	0	0.0	0–70.8
		Inpatient secondary	0	N/A	N/A	N/A
		Emergency	24	1	4.2	0.1–21.1
		Outpatient	17	0	0.0	0.0–19.5
	Total		820	223	27.2	23.5–29.4

CI indicates confidence interval; *ICD, International Classification of Diseases*; N/A, not available; PPV, positive predictive value; PSVT, paroxysmal supraventricular tachycardia.

Most of the 223 confirmed symptomatic PSVT episodes were classified as unspecified PSVT (65.5%), with the remainder being specific PSVT subtypes based on available diagnostic information: preexcitation syndrome/Wolff Parkinson White syndrome (14%), atrioventricular nodal reentry tachycardia (8.1%), ectopic atrial tachycardia (4.9%), multifocal atrial tachycardia (2.2%), atrioventricular reentry tachycardia (1.3%), atrioventricular junctional tachycardia (1.3%), and other specified cardiac dysrhythmias (1.8%).

### Period Prevalence of PSVT

We estimated the period prevalence of symptomatic PSVT in the KPNC population to be 140 per 100 000 members (95% CI, 100–179) based on the subset of diagnostic code/setting combinations that met our minimum criteria in terms of PPV and frequency of occurrence (Figure). The prevalence significantly differed across patient subgroups. Prevalence of symptomatic PSVT was substantially higher with older age, with a >20‐fold higher prevalence for participants aged ≥65 years compared with those aged 18 to 24 years (Table 2). Symptomatic PSVT prevalence was also higher in women than in men and in individuals who were black or white compared with Asian/Pacific Islanders (Table 2). A higher prevalence of symptomatic PSVT was also observed among patients with chronic kidney disease, diabetes mellitus, hypertension, coronary heart disease, and heart failure (Table 2).

**Figure 1:**
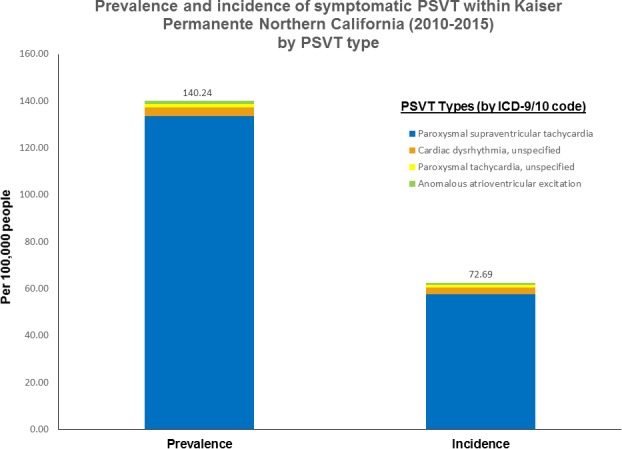
Prevalence and incidence of symptomatic paroxysmal supraventricular tachycardia (other than atrial fibrillation or atrial flutter) within Kaiser Permanente Northern California (2010–2015).

**Table 2:** Prevalence and Incidence of Symptomatic PSVT Episodes by Patient Subgroup (2010–2015)

	Period Prevalence Per 100 000 (95% CI)	Incidence Per 100 000 (95% CI)
Overall	140.2 (100.1–179.2)	72.7 (52.5–91.8)
Age, y		
18–24	23.3 (16.7–29.8)	14.8 (10.6–18.8)
25–44	59.1 (42.9–74.4)	34.1 (25.2–42.2)
45–64	177.5 (127.2–225.9)	96.7 (70.2–121.4)
≥65	479.6 (338.3–618.9)	231.9 (164.7–297.6)
Sex		
Men	110.1 (78.1–141.4)	61.9 (44.3–78.7)
Women	169.1 (121.0–215.4)	83.1 (60.4–104.3)
Race		
White	192.3 (136.7–246.6)	99.75 (71.64–126.6)
Black	219.9 (157.7–279.2)	106.8 (77.72–133.4)
Asian/Pacific Islander	92.7 (66.6–117.9)	51.48 (37.62–64.45)
Chronic kidney disease		
Yes	573.3 (405.6–736.2)	266.4 (189.1–341.2)
No	124.9 (89.2–159.5)	66.5 (48.1–83.8)
Diabetes mellitus		
Yes	373.9 (265.9–478.2)	183.4 (131.6–232.5)
No	123.6 (88.2–158.0)	66.4 (48.0–83.9)
Hypertension		
Yes	415.9 (294.2–534.9)	193.7 (138.6–46.5)
No	94.9 (68.1–120.7)	53.9 (39.1–67.8)
Coronary heart disease		
Yes	697.0 (494.4–891.5)	298.7 (212.5–381.3)
No	157.3 (112.2–201.0)	71.3 (51.5–90.0)
Heart failure		
Yes	825.0 (579.4–1064.7)	365.9 (256.3–473.6)
No	154.4 (110.3–197.3)	70.5 (51.0–89.0)

CI indicates confidence interval.

### Incidence of PSVT

We estimated the incidence of symptomatic PSVT episodes after excluding patients with evidence of having had diagnosed PSVT before entering the cohort. The incidence of presumed new symptomatic PSVT episodes between 2010 and 2015 was 73 per 100 000 (95% CI, 53–92), with variations among patient subgroups similar to those observed for prevalence during the study period (Table 2).

### National Burden of PSVT

Based on the age‐ and sex‐adjusted prevalence estimates found in KPNC, we estimate the US prevalence of symptomatic PSVT in 2010 to be 168 per 100 000 (95% CI, 120–215). This translated to an estimated 393 810 (95% CI, 280 489–503 998) affected adult Americans.

## Discussion

In a large, diverse, community‐based population receiving care within an integrated healthcare delivery system, we found several combinations of diagnosis codes and care settings to have acceptable predictive value for identifying valid symptomatic, sustained PSVT episodes. We estimated a prevalence of 140 per 100 000 and an incidence of 73 per 100 000 in our study population, with notably higher burden in older people, women, those of white or black race, and those with cardiovascular disease (coronary disease or heart failure) or major cardiovascular risk factors (chronic kidney disease, diabetes mellitus, or hypertension). Furthermore, we projected that at least 393 810 Americans experience symptomatic, sustained PSVT.

We found high PPV for the specific PSVT codes (427.0 in *ICD‐9* and I47.1 in *ICD‐10*) in the emergency department because most of these encounters included ECG confirmation of sustained regular narrow‐complex tachycardia that then reverted spontaneously or after specific treatment (eg, intravenous adenosine). The PPV of *ICD‐9* code 426.7 for anomalous atrioventricular conduction was also high in the emergency department setting, where it appears to identify episodes of acute, sustained, symptomatic PSVT. Among inpatients, a primary diagnosis of PSVT also appears to have higher PPV because patients were admitted for specific treatment. A secondary diagnosis of PSVT had substantially lower PPV than a primary diagnosis in the inpatient setting. Finally, all codes for PSVT in the outpatient setting had low PPV, probably reflecting a “problem list” diagnosis of uncertain reliability rather than a specific encounter for a symptomatic arrhythmia. Codes for palpitations, atrial premature contractions, unspecific tachycardias, and other specified cardiac arrhythmias all had low PPV for identifying symptomatic PSVT in all care settings. Consequently, it is important to consider the setting of care as well as the diagnostic code for reliable identification of symptomatic PSVT episodes.

Very limited data exist about the epidemiology of PSVT in the general US population. In the Marshfield Epidemiologic Study Area, potential cases of PSVT between 1979 and 1993 in Wisconsin were identified using selected *ICD‐9* codes (426.7, 427.0, 427.2, 427.6, 427.9, 785.0, 785.1) found in databases from the Marshfield Clinic.^2^ Among 2223 potential prevalent cases of PSVT, 600 patients had medical records and ECGs available, and 31 cases (PPV 5.2%) were confirmed and considered clinically significant based on having associated symptoms or requiring specific acute or long‐term therapy. Based on the weighted sampling design, the Marshfield investigators estimated the prevalence of PSVT to be 225 per 100 000 people, with higher prevalence in those aged ≥65 years (616 per 100 000) versus <65 years (165 per 100 000) and in women (256 per 100 000) compared with men (194 per 100 000). Among 1163 people who had potential incident PSVT between July 1991 and June 1993, 33 were confirmed cases (PPV 2.8%) for an estimated incidence of 35 per 100 000 people. Finally, the authors extrapolated their findings to the 1990 US population and projected that PSVT affected ≈570 000 Americans, with an estimated 89 000 new cases per year.^2^ Although this study provided important insights into population‐based epidemiology of PSVT, it also highlighted the low PPV of the studied *ICD‐9* codes for clinically significant PSVT and was limited by the small sample size, restricted racial/ethnic diversity, lack of detail about the specific type of PSVT, and earlier study period that may not reflect contemporary epidemiology.

Murman et al reported on the frequency of emergency department visits for supraventricular tachycardia in the United States based on data between 1993 and 2003 from the National Hospital Ambulatory Medical Care Survey.^11^ The occurrence of supraventricular tachycardia–related visits was based on *ICD‐9* codes 426.7 or 427.0 found in probability‐weighted samples of emergency department visits. The authors estimated that ≈50 000 visits in US emergency departments may be related to supraventricular tachycardia (estimated 18 visits per 100 000 US people; 95% CI, 140–230). The annual visit rate was higher for individuals aged ≥65 years (39 per 100 000) than for those aged <65 years (15 per 100 000) and higher for women (26 per 100 000) than for men (11 per 100 000).^11^ The study was limited by evaluating only encounters in the emergency department, use of administrative codes without medical records review to confirm the presence and duration of arrhythmia, the relatively small number of cases identified in their sample, and the use of data from an earlier time period that may not be fully generalizable to the current era.

Our study was strengthened by rigorous review of relevant medical records using standardized criteria to estimate the true PPV of available *ICD‐9* and *ICD‐10* diagnostic codes suggestive of potential symptomatic PSVT observed in different care settings. Our source population was large, ethnically diverse, and highly representative of the surrounding and statewide adult populations. We were also able to estimate PSVT burden across important patient demographic and clinical subgroups.

Our study also had several limitations. We focused on identification of any PSVT unrelated to atrial fibrillation or atrial flutter, but this includes a heterogeneous set of conditions, and we had limited available data and precision to examine more specific PSVT types. Given that we applied a minimum threshold for acceptable PPV and frequency for selecting the diagnostic code/care setting combinations, we may have missed cases of true symptomatic PSVT. This includes patients who had symptomatic PSVT but had spontaneous resolution before the arrhythmia was able to be confirmed. Patients with symptomatic PSVT but who did not seek out medical care also would not have been captured. We used diagnostic codes to exclude patients with atrial flutter, but given the potential for misclassification, we may have excluded patients who actually had a non–atrial flutter PSVT. Collectively, this would lead to our estimates being conservative. In contrast, the limited precision in the PPV for some of our included code/setting combinations may contribute to an overestimate in the actual burden of PSVT. Furthermore, in calculating incident PSVT, we searched up to 4 years before the index date, which may have overestimated the true incidence of PSVT. Our findings also may not be fully generalizable to other health systems, geographic areas, or uninsured people. Finally, it is important to emphasize that we identified episodes of care for sustained, symptomatic PSVT and not for individuals who may have had PSVT in the past but for whom it was not an active problem.

In summary, we provide new, contemporary information about the variable accuracy of diagnostic codes in different clinical care settings and updated estimates of the burden of symptomatic PSVT episodes within the United States. In addition, selected demographic and clinical characteristics can identify people who experience a higher burden of symptomatic PSVT.

## Sources of Funding

This work was supported by a research grant from Milestone Pharmaceuticals.

## Disclosures

Drs Go, Hlatky and Solomon have received research funding from Milestone Pharmaceuticals for this study. The remaining authors have no disclosures to report.
